# An easy-to-use nomogram for predicting in-hospital mortality risk in COVID-19: a retrospective cohort study in a university hospital

**DOI:** 10.1186/s12879-021-05845-x

**Published:** 2021-02-05

**Authors:** Hazal Cansu Acar, Günay Can, Rıdvan Karaali, Şermin Börekçi, İlker İnanç Balkan, Bilun Gemicioğlu, Dildar Konukoğlu, Ethem Erginöz, Mehmet Sarper Erdoğan, Fehmi Tabak

**Affiliations:** 1 Department of Public Health, Cerrahpasa Faculty of Medicine, Istanbul University-Cerrahpasa, Kocamustafapasa, Fatih, 34098 Istanbul, Turkey; 2 Department of Infectious Diseases, Cerrahpasa Faculty of Medicine, Istanbul University-Cerrahpasa, Kocamustafapasa, Fatih, 34098 Istanbul, Turkey; 3 Department of Pulmonary Diseases, Cerrahpasa Faculty of Medicine, Istanbul University-Cerrahpasa, Kocamustafapasa, Fatih, 34098 Istanbul, Turkey; 4 Department of Biochemistry, Cerrahpasa Faculty of Medicine, Istanbul University-Cerrahpasa, Kocamustafapasa, Fatih, 34098 Istanbul, Turkey

**Keywords:** COVID-19, Risk factor, Mortality, Nomogram, Fatal outcome

## Abstract

**Background:**

One-fifth of COVID-19 patients are seriously and critically ill cases and have a worse prognosis than non-severe cases. Although there is no specific treatment available for COVID-19, early recognition and supportive treatment may reduce the mortality. The aim of this study is to develop a functional nomogram that can be used by clinicians to estimate the risk of in-hospital mortality in patients hospitalized and treated for COVID-19 disease, and to compare the accuracy of model predictions with previous nomograms.

**Methods:**

This retrospective study enrolled 709 patients who were over 18 years old and received inpatient treatment for COVID-19 disease. Multivariable Logistic Regression analysis was performed to assess the possible predictors of a fatal outcome. A nomogram was developed with the possible predictors and total point were calculated.

**Results:**

Of the 709 patients treated for COVID-19, 75 (11%) died and 634 survived. The elder age, certain comorbidities (cancer, heart failure, chronic renal failure), dyspnea, lower levels of oxygen saturation and hematocrit, higher levels of C-reactive protein, aspartate aminotransferase and ferritin were independent risk factors for mortality. The prediction ability of total points was excellent (Area Under Curve = 0.922).

**Conclusions:**

The nomogram developed in this study can be used by clinicians as a practical and effective tool in mortality risk estimation. So that with early diagnosis and intervention mortality in COVID-19 patients may be reduced.

## Background

At the end of 2019, a group of similar pneumonia cases of unknown etiology in Wuhan City, China were reported. On 7 January 2020, a novel strain of coronavirus, now referred to as severe acute respiratory syndrome coronavirus 2 (SARS-CoV-2), was isolated. Later on 11 March 2020, COVID-19 was declared a pandemic by the World Health Organization (WHO) [1, 2]. As of January 6, 2021 there are 86.920.070 cases and 1.878.057 deaths worldwide [3].

The majority of COVID-19 patients experience mild to moderate respiratory illness and have no need for special treatment [4]. However, 20% of patients are severe and critically ill cases who are at risk of progressing to acute respiratory distress syndrome (ARDS), acute respiratory failure and/or multiple organ dysfunction [5]. Moreover, the risk of a fatal outcome is higher in patients with the severe form of the disease than the non-severe [6]. The case fatality ratio (CFR) of COVID-19 varies from less than 0.1% to over 25% by country but this ratio is as low as 0% in mild patients while it reaches 49% in critical cases [5, 7]. Although there is no specific treatment available for COVID-19, early recognition and supportive treatment of patients with a poor prognosis may reduce mortality [8].

COVID-19 disease has a more severe course especially in those of an older age and comorbidity such as hypertension, diabetes, coronary artery disease, cancer, and chronic renal failure [9]. In addition, previous studies have shown that COVID-19-related deaths are more common in patients whose laboratory parameters such as neutrophil, D-dimer, lymphocyte, interleukin 6 (IL-6), aspartate aminotransferase (AST) are not within the normal range [8, 10, 11]. As the right and immediate decision making of treatment strategies may decrease the risk of mortality it is urgent that the risk factors associated with fatal outcomes be identified. A number of risk factors have been identified with previous studies, but no consensus has been reached and risk factors remain uncertain [11]. In addition, some of the risk factors found in previous studies (such as IL-6, Glasgow Coma Scale and viral load) are not controlled for every patient in their daily routine. Therefore, a nomogram that includes parameters like routine biochemistry tests would be more practical and cost-effective for predicting risk of mortality. In addition, since variables are not categorized in nomograms developed in some previous studies, it is difficult to calculate the risk manually and it is not practical for clinicians.

## Methods

The aim of this retrospective study is to develop a functional nomogram that can be used by clinicians to estimate the risk of in-hospital mortality in patients hospitalized and treated for COVID-19 disease, and to compare the accuracy of model predictions with previous nomograms.

### Study design and participants

This retrospective study enrolled 709 of 920 patients who were over 18 years old and received inpatient treatment for COVID-19 disease at Cerrahpasa Faculty of Medicine (Istanbul / Turkey) between March 16, 2020 and June 18, 2020. Oropharyngeal and nasal swab samples were taken from all patients upon hospital admission [12]. A positive result of real time reverse transcriptase polymerase chain reaction of swabs was defined as a confirmed COVID-19 case [13]. For polymerase chain reaction (PCR) negative COVID-19 patients, chest computed tomography (CT) is a sensitive diagnostic approach in the early term and recommended for faster triage of those patients. The patients with negative PCR results but typical chest CT findings such as peripheral, bilateral, or multifocal round ground-glass opacity, received treatment for COVID-19 if the clinical manifestation could not be explained by another cause / disease [12]. Among the 709 cases, 204 had typical chest CT findings while PCR test was negative. Two hundred eleven patients treated for COVID-19 were not included in this study because they had neither PCR positivity nor typical thorax CT findings.

Inclusion criteria: To receive inpatient treatment for COVID-19 disease and to be over age of 18 (920 patients were included).

Exclusion criteria: To have neither PCR positivity nor typical thorax CT findings (211 patients were excluded).

### Data collection and laboratory procedures

The demographic (age, sex, and comorbidity), clinical (SpO_2_ at admission and symptoms), and laboratory parameters, and the final outcomes (dead/alive) of the patients were acquired from the medical records. The symptoms included: fever, cough, and the dyspnea which is a subjective awareness of the sensation of uncomfortable breathing. The laboratory parameters involved: hematocrit (HCT), neutrophil-lymphocyte ratio (NLR), C-reactive protein (CRP), urea, creatinine, aspartate aminotransferase (AST), alanine aminotransferase (ALT), lactate dehydrogenase (LDH), ferritin, fibrinogen, and D-dimer. Only the initial laboratory results of the patients were assessed.

Blood samples were centrifuged at 3500 rpm for 10 min for biochemical analysis. Blood gas analyzes were performed within 30 min, and all other routine analyzes were performed within 2 h at the latest without waiting in the laboratory. Complete blood count and determination of leukocyte subgroups were performed on Beckman Coulter LH 780 analyzer. The enzymatic kinetic method was used for serum AST and LDH analyzes; the colorimetric photometric method for urea and creatinine analysis; and the immune turbidimetric method for CRP analysis (Roche diagnostic, cobas 8000 modular analyzer). D-Dimer and fibrinogen analyzes were performed on the Sysmex CS2500 hemagglutinin analyzer. Ferritin analyzes were determined by electrochemiluminescence method (Roche diagnostic, cobas 8000 modular analyzer). Blood gas analysis was performed on Radiometer ABL 800 devices.

### Statistical analysis

Statistical analysis was performed using SPSS v21.0 (SPSS Inc., Chicago, IL, USA) and Microsoft Office Excel (Microsoft Corporation, Redmond, WA, USA). Normality was evaluated using the Kolmogorov Smirnov Test. Continuous variables were presented as mean ± standard deviation, categorical variables as frequency and percentage. Categorical variables were analysed using the Chi-square test or the Fisher Exact test, where appropriate. Continuous variables were analysed using the Student t test. Laboratory results and oxygen saturation (SpO2) were categorized using the Receiver Operating Characteristic (ROC) curve, and the point where the sensitivity and specificity were maximized was selected as cut-off point. Two cut-off points were selected when the distribution was multimodal. Logistic Regression analysis was performed with variables that are significant in the univariate analysis. Odds ratios (OR) were adjusted for age group and sex, except the age group only adjusted for sex. Comorbidities that are significant in the adjusted analysis cancer, heart failure and chronic renal failure, were assembled as a new variable named “specific comorbidity”, and the OR of these specific comorbidities were given. Multivariable Logistic Regression analysis by using the backward method was performed to assess the possible predictors of a fatal outcome. To avoid possible multicollinearity, only one of the highly correlated variables, the one with a high contribution to the model, was included in the multivariable logistic regression analysis. Results were presented as OR and 95% Confidence Intervals (95% CI). Each category of the variables in nomogram were given a point by rounding ORs to the nearest integer, and total points were calculated by summing these points. ROC curve was used to assess the accuracy of model predictions, and area under curve (AUC) was given. The predicted probability of a fatal outcome was determined using Logistic regression analysis. Three different models were generated for sensitivity analysis and accuracy of these model predictions were evaluated. Patients were divided into six groups according to their scores (1st-4th quintiles, 90th percentile, and 95th percentile) then, cox regression analysis and Log Rank test was performed to obtain survival curves and difference in survival. A *p*-value < 0.05 was accepted for statistical significance.

## Results

### Characteristics of the patients

Of the 709 patients treated for COVID-19, 75 (11%) died due to complications related to COVID-19 disease and 634 (89%) survived. The mean follow-up time was 9 ± 7 days. The mean age of the patients with a fatal outcome was 69 ± 14, and it was significantly higher than the mean age of the patients who survived. (*p* < 0.001). There was no significant difference in gender between the two groups (*p* = 0.393), 47% of the survivors and 41% of the non-survivors were female. The most frequent symptoms in the cohort were fever (33%), cough (42%), and dyspnea (31%). Dyspnea existed in 48% of the patients with a fatal outcome which was significantly more than the survivors (*p* = 0.001). The prevalence of hypertension, diabetes mellitus, cancer, heart failure, and chronic renal failure was significantly higher in the patients who died (*p* = 0.002; *p* = 0.001; *p* < 0.001; *p* < 0.001; *p* < 0.001, respectively) whereas the prevalence of asthma and/or chronic obstructive pulmonary disease (COPD) and coronary artery disease was higher but not significant (*p* = 0.127; 0.088, respectively). SpO2 at admission was different between the groups (*p* < 0.001). SpO2 was 88% or less in 34% of the patients who died while it was 88% or less in 7% of the survivors. All of the initial laboratory parameters were statistically different between the two groups (*p* < 0.05). The demographic, clinical, and initial laboratory parameters of the patients are given in Table 1.

**Table 1:** The demographic, clinical, and initial laboratory parameters of the patients

Parameters	Non-survivor (***n*** = 75)	Survivor (***n*** = 634)	***p***-value
**Age**, mean ± SD	69 ± 14	55 ± 15	< 0.001^†^
**Sex (Female)**, n (%)	31 (41)	295 (47)	0.393^ǂ^
**Age group**, n (%)			< 0.001^ǂ^
19–54	10 (13)	327 (52)	
55–64	19 (25)	149 (24)	
65–74	20 (27)	85 (13)	
≥ 75	26 (35)	73 (12)	
**Comorbidities**, n (%)			
Hypertension	29 (39)	143 (23)	0.002^ǂ^
Diabetes Mellitus	19 (25)	73 (12)	0.001^ǂ^
Cancer	24 (32)	24 (4)	< 0.001^ǂ^
Heart failure	11 (15)	21 (3)	< 0.001^ǂ^
Asthma/COPD	11 (15)	58 (9)	0.127^ǂ^
Coronary artery disease	9 (12)	42 (7)	0.088^ǂ^
Chronic renal failure	11 (15)	19 (3)	< 0.001^ǂ^
**Symptoms**, n (%)			
Fever	21 (28)	212 (33)	0.343^ǂ^
Cough	16 (21)	280 (44)	< 0.001^ǂ^
Dyspnea	36 (48)	181 (29)	0.001^ǂ^
**SpO**_**2**_ **at admission**, n (%)			< 0.001^ǂ^
≥ 95%	16 (22)	375 (63)	
89–94%	32 (44)	182 (30)	
≤ 88%	25 (34)	41 (7)	
**Initial laboratory results**, n (%)			
**HCT**			< 0.001^ǂ^
> 35%	24 (32)	472 (75)	
≤ 35%	50 (68)	160 (25)	
**NLR**			< 0.001^ǂ^
< 2.95	12 (17)	362 (57)	
≥ 2.95	60 (83)	272 (43)	
**CRP**			< 0.001^ǂ^
≤ 50 mg/L	13 (18)	439 (69)	
> 50 - < 100 mg/L	14 (19)	108 (17)	
≥ 100 mg/L	47 (64)	86 (14)	
**Urea**			< 0.001^ǂ^
≤ 40 mg/dL	26 (35)	493 (78)	
> 40 - < 75 mg/dL	24 (32)	110 (17)	
≥ 75 mg/dL	24 (32)	30 (5)	
**Creatinine**			< 0.001^ǂ^
≤ 1.5 mg/dL	52 (70)	585 (92)	
> 1.5 - < 3 mg/dL	10 (14)	40 (6)	
≥ 3 mg/dL	12 (16)	8 (1)	
**AST**			< 0.001^ǂ^
≤ 40 IU/L	37 (49)	530 (84)	
> 40 - < 80 IU/L	17 (23)	87 (14)	
≥ 80 IU/L	21 (28)	17 (3)	
**ALT**			< 0.001^ǂ^
≤ 40 IU/L	50 (68)	534 (84)	
> 40 - < 80 IU/L	14 (19)	76 (12)	
≥ 80 IU/L	10 (14)	24 (4)	
**Ferritin**			< 0.001^ǂ^
≤ 400 ng/mL	17 (26)	446 (73)	
> 400 - < 1000 ng/mL	16 (24)	123 (20)	
≥ 1000 ng/mL	33 (50)	45 (7)	
**LDH**			< 0.001^ǂ^
≤ 400 IU/L	37 (49)	561 (89)	
> 400 - < 700 IU/L	26 (35)	62 (10)	
≥ 700 IU/L	12 (16)	10 (2)	
**Fibrinogen**			0.030^ǂ^
≤ 400 mg/dL	19 (27)	252 (43)	
> 400 - < 750 mg/dL	43 (61)	294 (50)	
≥ 750 mg/dL	8 (11)	41 (7)	
**D-dimer**			< 0.001^§^
≤ 4 mg/L	47 (65)	590 (96)	
> 4 - < 7 mg/L	8 (11)	12 (2)	
≥ 7 mg/L	17 (24)	16 (3)	

*SD* Standard deviation, *COPD* Chronic obstructive pulmonary disease, *SpO2* Oxygen saturation, *HCT* Hematocrit, *NLR* Neutrophil to lymphocyte ratio, *CRP* C-reactive protein, *AST* Aspartate aminotransferase, *ALT* Alanine aminotransferase, *LDH* Lactate dehydrogenase

^†^, Student t test; ^ǂ^, Chi-square test; ^§^, Fisher Exact test

### Association between patients’ characteristics and outcomes

In adjusted analysis, no significant difference was found between the female and male risk of death (*p* = 0.219). The risk of a fatal outcome was higher in elderly patients. The risk of death increased by 4.07 fold in the patients aged between 55 and 64 years old (95% CI 1.85–8.98; *p* = 0.001), 7.85 fold in the patients aged between 65 and 74 years old (95% CI 3.54–17.43; *p* < 0.001) and 11.95 fold in the patients aged 75 years old and above (95% CI 5.51–25.93; *p* < 0.001). The patients with hypertension, diabetes mellitus, asthma/COPD or coronary artery disease didn’t have an increased risk of death, while the patients with cancer, heart failure or chronic renal failure had an increased risk (*p* = 0.337, *p* = 0.064, *p* = 0.576, *p* = 0.963, *p* < 0.001, *p* = 0.006, *p* = 0.012, respectively; Data not presented in table). Patients having at least one of the comorbidities cancer, heart failure or chronic renal failure were referred to as patients with specific comorbidities. The specific comorbidity, dyspnea, SpO2, and all initial laboratory parameters except fibrinogen increased the risk of death by 2.19 to 20.78 fold. The results of the adjusted analysis are presented in Table 2.

**Table 2:** Odds ratio and 95% confidence interval for the risk of fatal outcome

	Adjusted^b^ analysis		Multivariable analysis		Point
Parameters	OR (95% CI)	***p*** value	OR (95% CI)	***p*** value	
Age group (Ref: 19–54)					
55–64	4.07 (1.85–8.98)	0.001			4
65–74	7.85 (3.54–17.43)	< 0.001			8
≥ 75	11.95 (5.51–25.93)	< 0.001			12
Specific comorbidity^a^ (Ref: Absence)	6.76 (3.89–11.75)	< 0.001	2.73 (1.28–5.84)	0.010	3
Dyspnea (Ref: Absence)	2.19 (1.32–3.64)	0.002	2.09 (1.04–4.20)	0.038	2
SpO_2_ at admission (Ref: ≥95%)					
89–94%	3.65 (1.92–6.95)	< 0.001	2.81 (1.23–6.43)	0.014	3
≤ 88%	11.18 (5.31–23.53)	< 0.001	8.81 (3.47–22.42)	< 0.001	9
Urea (Ref: ≤40 mg/dL)					
> 40 - < 75 mg/dL	2.3 (1.20–4.43)	0.013			
≥ 75 mg/dL	7.85 (3.69–16.71)	< 0.001			
Creatinine (Ref: ≤2.5 mg/dL)					
> 2.5 - < 3 mg/dL	4.31 (0.87–21.33)	0.073			
≥ 3 mg/dL	12.14 (4.38–33.64)	< 0.001			
HCT (Ref: > 35%)	5.16 (2.95–9.02)	< 0.001	3.07 (1.50–6.28)	0.002	3
NLR (Ref: < 2.95)	4.78 (2.46–9.27)	< 0.001			
CRP (Ref: ≤50 mg/L)					
> 50 - < 100 mg/L	4.06 (1.82–9.06)	0.001	1.5 (0.55–4.11)	0.431	2
≥ 100 mg/L	12.75 (6.44–25.26)	< 0.001	5.67 (2.48–12.95)	< 0.001	6
AST (Ref: ≤40 IU/L)					
> 40 - < 80 IU/L	2.69 (1.39–5.19)	0.003	1.97 (0.85–4.55)	0.113	2
≥ 80 IU/L	15.62 (7.13–34.24)	< 0.001	6.81 (2.45–18.09)	< 0.001	7
ALT (Ref: ≤40 IU/L)					
> 40 - < 80 IU/L	2.36 (1.19–4.68)	0.014			
≥ 80 IU/L	4.54 (1.87–10.94)	0.001			
Ferritin (Ref: ≤400 ng/mL)					
> 400 - < 1000 ng/mL	3.69 (1.73–7.88)	0.001	1.5 (0.62–3.65)	0.368	2
≥ 1000 ng/mL	20.78 (9.83–43.93)	< 0.001	4.72 (2.05–10.91)	< 0.001	5
LDH (Ref: ≤400 IU/L)					
> 400 - < 700 IU/L	5.19 (2.80–9.64)	< 0.001			
≥ 700 IU/L	19.44 (7.26–52.02)	< 0.001			
Fibrinogen (Ref: ≤ 400 mg/dL)					
> 400 - < 750 mg/dL	1.57 (0.87–2.82)	0.136			
≥ 750 mg/dL	1.97 (0.76–5.13)	0.164			
D-dimer (Ref: ≤4 mg/L)					
> 4 - < 7 mg/L	5.39 (1.98–14.64)	0.001			
≥ 7 mg/L	10.12 (4.55–22.52)	< 0.001			

*Ref* Reference category, *SpO*_*2*_ Oxygen saturation, *HCT* Hematocrit, *NLR* Neutrophil to lymphocyte ratio, *CRP* C-reactive protein, *AST* Aspartate aminotransferase, *ALT* Alanine aminotransferase, *LDH* Lactate dehydrogenase.

^a^ Having at least one of the following comorbidities: cancer, heart failure, chronic renal failure

^b^ Adjusted for age group and sex, except the age group only adjusted for sex

**Fig. 1:**
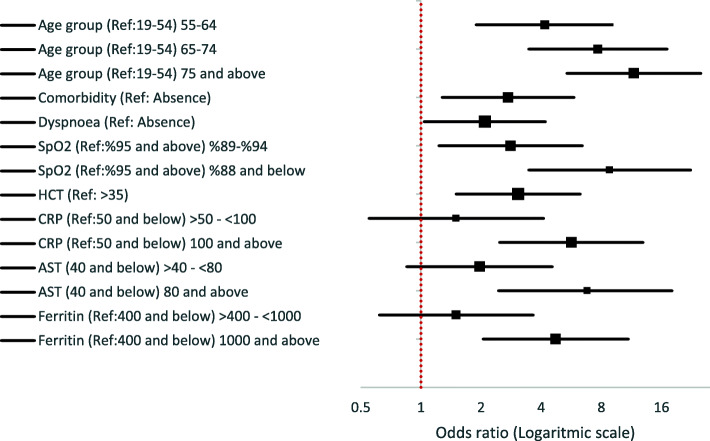
Forest plot showing results of multivariable regression analysis

A total of 13 parameters that are associated with a fatal outcome, and the sex were included in multivariable logistic regression analysis. The results showed that the specific comorbidities, dyspnea, SpO2, HCT, CRP, AST and ferritin were the possible predictors of the fatal outcome in inpatients. The risk of death was increased by 2.73 fold (95% CI 1.28–5.84; *p* = 0.010) in the patients with specific comorbidities. Having dyspnea increased the risk of a fatal outcome by 2.09 fold (95% CI 1.04–4.20; *p* = 0.038). Also, having SpO2 less than 95% increased the risk of death. SpO2 between 89 and 94% increased the risk by 2.81 fold (95% CI 1.23–6.43; *p* = 0.014), while SpO2 equal or less than 88% increased the risk by 8.81 fold (95% CI 3.47–22.42; *p* < 0.001). The mortality risk of patients whose HCT equal or less than 35% was increased by 3.07 fold (95% CI 1.50–6.28; *p* = 0.002). The risk of death increased by 5.67 fold (95% CI 2.48–12.95; *p* < 0.001) in patients whose CRP was equal or more than 100 mg/L, 6.81 fold (95% CI 2.45–18.09; *p* < 0.001) in the patients whose AST was equal or more than 80 IU/L, and 4.72 fold (95% CI 2.05–10.91; *p* < 0.001) in the patients whose ferritin was equal or more than 1000 ng/mL. The increase in mortality risk was not statistically significant in patients whose CRP was between 50 and 100 mg/L, AST is between 40 and 80 IU/L, and fibrinogen is between 400 and 1000 ng/mL. The results of the adjusted analysis are presented in Table 2 and Fig. 1.

### Construction of mortality prediction nomogram

**Fig. 2:**
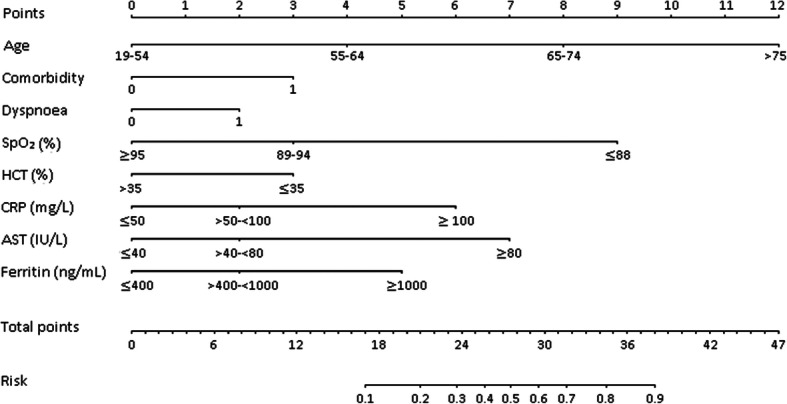
Nomogram predicting the risk of fatal outcome

In order to predict the mortality risk of inpatients, a nomogram based on seven parameters that were significant in multivariable analysis and the age group was constructed. The age group and the intermediate category of CRP, AST, and ferritin was not significant in multivariable analysis, however they were included in the nomogram as they are clinically important. The point of each parameter was given in Table 2. The total point that varies from zero to 47 was calculated by summing the point taken from each parameter. The risk of death by total points were shown in the nomogram. The risk of death was lower than 10% for those below 17 points, and higher than 90% for those with over 38 points. The nomogram is shown in Fig. 2.

### The accuracy of model prediction and the comparison with previous nomograms

**Fig. 3:**
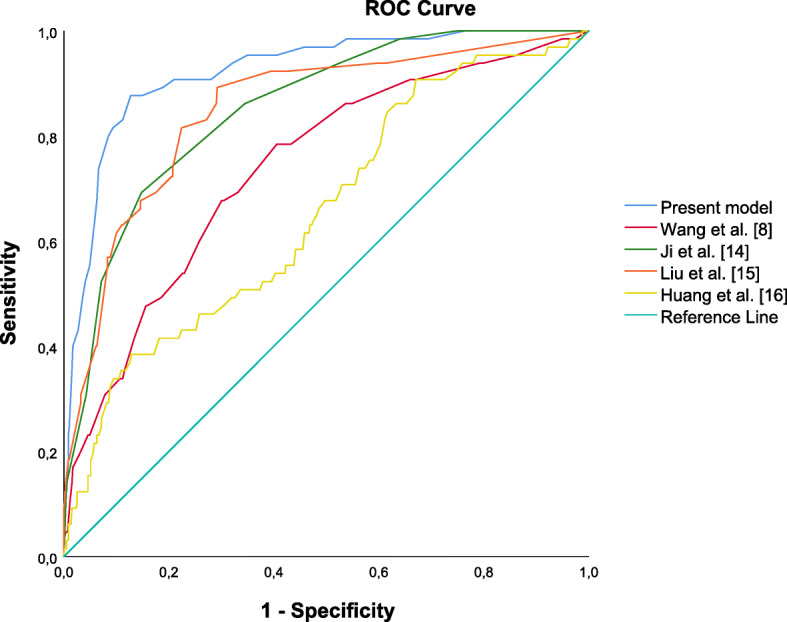
ROC curves of present study and a number of previous studies

The prediction ability of total points was excellent. The AUC was 0.92 (95% CI 0.89–0,96; *p* < 0.001). The predictive ability of the nomogram was compared with previous nomograms that could be applied to the current data. Four nomograms predicting mortality risk or disease progression were evaluated using the data of the present cohort. The results are given in Fig. 3 and Table 3.

**Table 3:** The accuracy of model prediction and the comparison with previous nomograms

Study	Area Under Curve	Standard Error	***p*** value	95% CI
Present model	0.922	0.017	< 0.001	0.888–0.955
Wang et al. [8]	0.739	0.033	< 0.001	0.675–0.804
Ji et al. [14]	0.852	0.023	< 0.001	0.807–0.897
Liu et al. [15]	0.850	0.026	< 0.001	0.799–0.902
Huang et al. [16]	0.655	0.036	< 0.001	0.584–0.725

### The sensitivity analysis

A sensitivity analysis was performed to assess the predictive ability and variability of the model in different groups of patients. Three different models were set, and all three models were statistically significant. In model 1, patients were divided into two groups according to the PCR results. The AUC of PCR negative patients was 0,94 (95% CI 0,91-0,98; *p* < 0.001), and the AUC of PCR positive patients was 0,92 (0,88-0,97; *p* < 0.001). In model 2, patients in the groups were randomly selected. The AUC of groups 1 and group 2 was 0,93 (95% CI 0,89-0,97; *p* < 0.001) and 0,92 (95% CI 0,87-0,96; *p* < 0.001), respectively. Groups in model 3 were determined according to patients’ date of hospital admission. The AUC of groups 1 and group 2 was 0,92 (95% CI 0,88-0,96; *p* < 0.001) and 0,95 (95% CI 0,91-0,98; *p* < 0.001), respectively. The results of the sensitivity analysis are presented in Table 4.

**Table 4:** Sensitivity analysis of nomogram

	Area Under Curve	Standard Error	***p*** value	95% CI
**Model 1**				
Group 1	0.944	0.018	< 0.001	0.909–0.978
Group 2	0.922	0.023	< 0.001	0.877–0.967
**Model 2**				
Group 1	0.932	0.021	< 0.001	0.890–0.974
Group 2	0.915	0.025	< 0.001	0.866–0.964
**Model 3**				
Group 1	0.918	0.021	< 0.001	0.877–0.959
Group 2	0.948	0.019	< 0.001	0.911–0.984

Model 1: Two group according to PCR result. Group 1, PCR negative; Group 2, PCR positive.

Model 2: Two group randomly divided.

Model 3: Two group according to date of hospital admission. Group 1, First 7 weeks; Group 2, Eight to fourteen weeks

### Survival and calibration curves

**Fig. 4:**
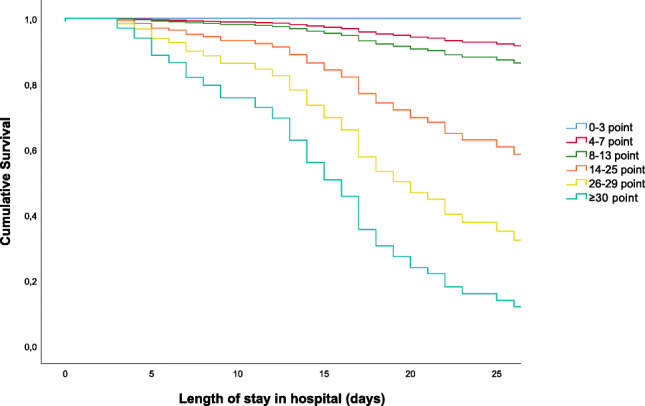
Survival curves of six group (1st-4th quintiles, 90th percentile, and 95th percentile)

Survival was significantly lower in patients with higher scores (Log Rank *p* < 0.001). Figure 4 shows survival curves of each of the groups.

**Fig. 5:**
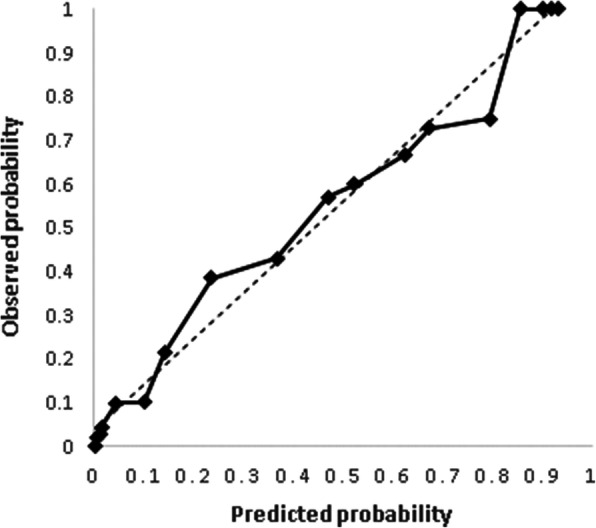
Observed versus predicted probability of in-hospital mortality (calibration; straight line)

Observed versus predicted probability of in-hospital mortality was shown in Fig. 5. The model showed good calibration (*R*^2^ = 0.985).

## Discussion

This study showed that age, certain comorbidities (cancer, heart failure, chronic renal failure), dyspnea, lower SpO2 and the laboratory parameters HCT, CRP, AST and Ferritin were independent risk factors associated with a fatal outcome. This result has some similarities but also differences to previous studies. Although there are differences in categorization, age was found to be a risk factor for mortality in previous studies [6, 8, 10, 11, 17–19]. A multicenter study showed that the risk of mortality increased by 18% in patients aged 70 or older [10]. In another study that developed a risk score to predict intensive care unit (ICU) admission and mortality in COVID-19 patients the risk of mortality was increased 4.90 fold in patients aged older than 63 [18]. In our study, age was significant when adjusted only for sex but it was not significant in multivariable analysis. In adjusted analysis, the risk of mortality was increased 4.07 fold in patients aged between 55 and 64, 7.85 fold in patients aged between 65 and 74, and 11.95 fold in patients aged 75 or older. There is another study where age was not found to be a risk factor for mortality. As mentioned in this study, the reason why age is not a risk factor for mortality may be the fact that rather than aging itself the age-related comorbidities affect mortality [11]. In our study, even if age was not a risk factor in multivariable analysis it was included in the nomogram as it has clinical importance.

In previous studies, comorbidity was found to be a risk factor for mortality. In a study assessing association between the presence of comorbidity and mortality, the hazard ratio was found to be higher in patients with existing chronic diseases such as hypertension, diabetes, and kidney disease [19]. There are also studies using the number of comorbidities while evaluating the relation between comorbidity and mortality. In the 4C Mortality Score developed by International Severe Acute Respiratory and Emerging Infection Consortium, the total score increases by 1 point if patients have one comorbidity but by 2 points if patients have two or more comorbidities [20]. On the other hand, there are some studies examining the effects of different comorbidities on mortality separately. Chen et al. found that coronary heart disease and cerebrovascular disease are independent risk factors for mortality [11]. In another study, it was found that the risk of a fatal outcome increased 9.23 and 33.48 fold in patients having chronic obstructive pulmonary disease or a history of heart failure, respectively [18]. Contrary to this, in early reports the prevalence of chronic respiratory conditions was low in patients admitted to hospital [21]. In a study that used both the number of comorbidity and cancer history as a risk factor for critical illness, one-unit increase in comorbidity increased the risk by 1.6 fold. Also, having cancer history increased the risk by 4.1 fold in same study [22]. In our study, asthma/COPD was not a risk factor for mortality whereas having at least one of the comorbidities cancer, heart failure, chronic renal failure was a risk factor, and increased the mortality by 2.73 fold.

Dyspnea is one of the serious symptoms seen in COVID-19 patients [4]. In the present study, almost half of the patients who died had dyspnea upon hospital admission. Dyspnea was one of the risk factors for a fatal outcome and it increased the risk of mortality by 2.09 fold. This result is consistent with similar studies. Chen et al. found that the hazard ratio of patients having dyspnea was 3.96 [11]. In a systematic review, patients having shortness of breath / dyspnea have a higher risk of critical illness and mortality with the OR of 4.16 [23]. In addition to dyspnea, low oxygen saturation was also a risk factor for mortality in our study. The risk of mortality increased by 2.81 fold in patients having SpO2 between 89 and 94% and by 8.81 fold in patients having SpO2 88% or lower. In a study evaluating the association between hypoxemia and mortality in patients with COVID-19, it was found that for each one-unit increase in SpO2, the mortality risk decreased by approximately 8%. When SpO2 was also analyzed categorically in the same study (≤90% vs > 90%), an SpO2 value of 90% or less was strongly associated with death (HR 47.41; 95% CI 6.29–357.48) [24]. In some patients, hypoxemia would worsen and ARDS could develop with the progression of the disease. ARDS occurs in 42% of patients with COVID-19 pneumonia, and 61–81% of patients requiring intensive care [25]. Dyspnea and hypoxemia suggest poor lung functionality and a lack of oxygen, and help early recognition of ARDS [23, 25]. Therefore, appropriate intervention in patients having dyspnea and hypoxemia can reduce the development of ARDS and the risk of mortality.

In this study, CRP is one of the risk factors for a fatal outcome in COVID-19 patients. The mortality risk of patients having a CRP level equal or more than 100 mg/L is 5.67 times higher than patients having a CRP level of 50 mg/L or less. The patients with a CRP level between 50 and 100 mg/L also have a higher risk of death but it is not statistically significant. However, it was included in the scoring because it is clinically valuable. CRP is an acute-phase protein induced mostly by IL-6 in response to inflammatory conditions [26]. As in the Severe Acute Respiratory Syndrome (SARS) and Middle East Respiratory Syndrome (MERS) diseases the inflammatory response plays a crucial role, as it does in COVID-19 disease [8]. Cytokine storm is critical to the progression of COVID-19 disease and it seems to be a significant cause of death [17, 27]. Similar to our study, Liu et al. found that patients with CRP levels higher than 41.8 mg/L are more likely to develop a severe form of the disease [27]. Also, Ruan et al. showed that the CRP level of patients who died is higher than patients who are discharged [17].

In our model, ferritin is another inflammatory marker used to predict the mortality risk. The COVID-19 patients who died had higher ferritin levels at the time of admission. A ferritin level of 1000 ng/mL and above increased the risk of death by 4.72 fold. The increase in risk of patients having a ferritin level between 400 and 1000 ng/mL was not significant but since it is clinically important, it was included in the model. As in CRP synthesis, ferritin synthesis can be induced by increased IL-6 during cytokine storm. Also macrophages might be responsible for ferritin production and lead to hyperferritinemia. While there are many studies on CRP level in COVID-19 patients, studies on ferritin are limited [28]. In a study assessing risk factors associated with ARDS in COVID-19 patients, ferritin is found to be a risk factor for ARDS but not a risk factor for death [25]. In another study, higher serum ferritin levels were associated with higher odds of death at the univariate analysis [29]. There are also a few studies reporting higher serum ferritin levels in severe patients and non-survivors [29–31]. Moreover, it is reported that as patients recovered the ferritin and IL-6 levels decreased. These findings may be evidence that hyperferritinemia is associated with inflammatory processes in COVID-19 disease [28]. Therefore, ferritin can be a useful parameter in predicting the disease severity and the mortality risk.

AST levels higher than 40 U/L were also associated with increased risk of death in our model. The mortality risk was increased 6.81 fold in patients having AST level of 80 U/L or higher. The risk is also higher in patients having AST levels between 40 and 80 U/L but it is not statistically significant. However, it is included in the model because it is clinically important. In the nomogram model developed by Chen et al. it is found that elevated AST (> 40 U/L) levels increase the risk for death by 2.2 fold [11]. Another study showed that as the AST level increases, the mortality risk also increases [8]. Coronavirus mainly targets the respiratory system however, previous studies have shown evidence of damage also to other organs including the liver. Liver damage in COVID-19 patients may be associated with an organ-specific immune response. Also hypoxemia, systemic cytokine storm and medications can also cause liver damage [32]. It is reported that severe COVID-19 patients have AST-predominant elevation of liver enzymes on admission [32, 33]. This finding is consistent with the results of our study and indicates that AST is an important marker for predicting clinical outcomes.

Another laboratory parameter associated with mortality risk in our nomogram model is HCT. The mortality risk increased by 3.07 fold in patients having decreased HCT (≤35%). Similar to our study, Wang et al. found that patients in the severe group have significantly lower HCT than those in the moderate group. In the same study, lower red blood cell (RBC) and hemoglobin were also reported in the severe group [34]. Decreased production of RBCs as a result of suppression of the bone marrow by the antiviral response may cause low HCT in severe COVID-19 patients [35, 36].

The nomogram model in this study showed excellent discrimination with AUC = 0.922 [37]. Moreover, in sensitivity analysis the maximum difference in AUC between groups was 3%, in other words, the nomogram gave similar results in different groups. On the other hand, when we evaluate the models previously developed that can be performed with the current data, none of them were as predictive as the model developed in the present study [8, 14–16].

This study had some limitations. First, since it was not possible to reach the full medical history of the patients, data about comorbidities was based on the patients’ self-reporting, thus, it may lead to recall bias. In addition, possible confounding factors, the duration of the comorbidity and whether the patient received regular treatment were not available in the records, so they could not be included in the analyses. Second, an important potential risk factor, viral load, was not assessed in our study. However, the aim of this study was to create a model that would enable clinicians to predict mortality risk with easily accessible data, and the current model showed excellent discrimination even without viral load. Third, the dynamics such as models of care, availability of resources, predefined local criteria for hospital admissions etc. couldn’t assessed. However, in sensitivity analysis cohort was divided into two groups according to date of hospital admission (the 1-7th weeks of the pandemic/ the 8-14th weeks of the pandemic) and the nomogram model showed outstanding discrimination for both groups. Finally, these results were obtained using data from a single-center. Therefore, external validity of the model couldn’t be assessed. The dynamics associated with patients’ admissions, and so the parameters at admission evaluated in this study, may vary locally. The predictions of the model in different cohorts should be assessed for external validation.

## Conclusion

This study showed that age, certain comorbidities (cancer, heart failure, chronic renal failure), dyspnea, lower SpO2 and the laboratory parameters HCT, CRP, AST and Ferritin were potential risk factors associated with mortality. The nomogram developed in this study can be used by clinicians as a practical and effective tool in in-hospital mortality risk estimation. So that with early diagnosis and intervention mortality in COVID-19 patients may be reduced.

## Data Availability

The datasets generated and/or analysed during the current study are not publicly available due to Turkish Personal Data Protection Law no 6698 but are available from the corresponding author on reasonable request.

## Abbreviations

ALT: Alanine aminotransferase
ARDS: Acute respiratory distress syndrome
AST: Aspartate aminotransferase
AUC: Area Under Curve
CFR: Case fatality ratio
CI: Confidence Intervals
COPD: Chronic obstructive pulmonary disease
COVID-19: Coronavirus disease 2019
CRP: C-reactive protein
CT: Computed tomography
EDTA: Ethylenediaminetetraacetic acid
HCT: Hematocrit
ICU: Intensive care unit
IL-6: Interleukin 6
LDH: Lactate dehydrogenase
MERS: Middle East respiratory syndrome
NLR: Neutrophil-lymphocyte ratio
OR: Odds ratio
PCR: Polymerase chain reaction
RBC: Red blood cell
ROC: Receiver Operating Characteristic
SARS: Severe acute respiratory syndrome
SARS-CoV-2: Severe acute respiratory syndrome coronavirus 2
SpO2: Oxygen saturation
WHO: World Health Organization
